# Jaw Claudication Caused by Atherothrombotic External Carotid Artery Occlusion: A Case Report

**DOI:** 10.7759/cureus.43911

**Published:** 2023-08-22

**Authors:** Kotaro Kohara, Takakazu Kawamata

**Affiliations:** 1 Department of Neurosurgery, Tokyo Women's Medical University, Tokyo, JPN

**Keywords:** recanalization, conservative therapy, atherothrombotic occlusion, external carotid artery, jaw claudication

## Abstract

Jaw claudication is a common symptom of giant cell arteritis (GCA), although atherothrombotic external carotid artery (ECA) occlusion is also known to cause jaw claudication. The patient was a 75-year-old male who experienced severe right jaw pain while chewing solid food. Magnetic resonance (MR) angiography showed right ECA occlusion. Based on laboratory tests and contrast-enhanced computed tomography (CT) angiography, atherothrombosis, not GCA, was suspected to be the cause of jaw claudication. Following conservative therapy with cilostazol, the pain was gradually alleviated in two months, and subsequent MR angiography after four months showed blood flow in the stenosed right ECA. The symptom completely disappeared in six months. Based on a previous report, we expected that jaw claudication will be ameliorated due to the development of collateral supply; however, spontaneous ECA recanalization caused improvement of symptoms in this case.

## Introduction

Jaw claudication is a symptom caused by a reduction in blood flow in the masseteric and facial arteries, which are branches of the external carotid artery (ECA) that supply blood to the masseter and temporalis muscles [1].

The most common cause of jaw claudication is giant cell arteritis (GCA) [2]. Recently, some cases of jaw claudication after internal carotid artery (ICA) stenting that impaired ECA blood flow have also been reported [1,3,4]. Although flow reduction in the ECA and/or its branches, regardless of the cause, can be responsible for jaw claudication, atherothrombotic ECA occlusion causing jaw claudication is rare [5], with only a few reports about its time course.

We present a case of jaw claudication caused by atherothrombotic ECA occlusion, which was treated by conservative therapy, and review the literature.

## Case presentation

**Figure 1:**
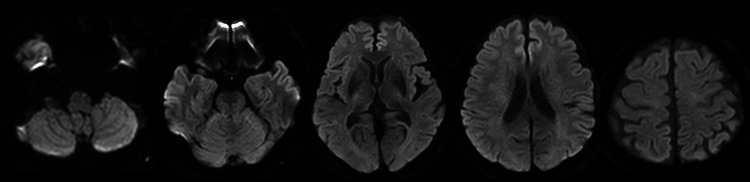
Diffusion-weighted magnetic resonance images showing no acute ischemic or hemorrhagic lesions

**Figure 2:**
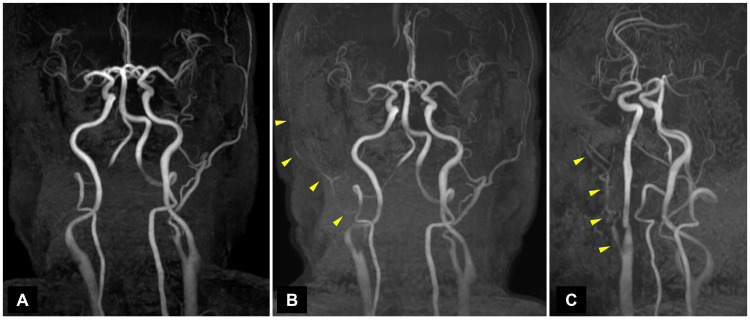
Cranio-cervical magnetic resonance angiograms showing occlusion of the right ECA at first (A) and right ECA recanalization and internal carotid artery stenosis four months later (B,C) ECA: external carotid artery

The patient was a 75-year-old male with jaw claudication that appeared without any prior warning signs. He experienced pain in his right jaw after chewing food a few times and could not chew subsequently. Therefore, he was unable to ingest solid food. He had a medical history of right microvascular decompression surgery for trigeminal neuralgia; however, the current pain was different from the previous stabbing pain associated with trigeminal neuralgia. He did not have other comorbidities such as hypertension or diabetes mellitus. He did not smoke or drink alcohol. The patient was conscious and ambulatory. No neurological deterioration including facial palsy, dysarthria, and sensory impairment was detected, except jaw claudication. His blood pressure was 135/112 mmHg. Blood examination did not show any abnormality, and the erythrocyte sedimentation rate (ESR) was 1 mm in an hour. Electrocardiography showed sinus rhythm, and no arrhythmia was detected on Holter electrocardiography. Magnetic resonance (MR) images showed no signs of cerebral stroke or abnormalities related to the symptom (Figure 1). MR angiography revealed right ECA occlusion from the carotid bifurcation, while no stenosis and major intracranial artery occlusions were detected (Figure 2). Contrast-enhanced computed tomography (CT) angiography showed no presence of arteritis, plaque, stenosis, or dissection from the aortic arch to the brachiocephalic trunk and right common carotid artery.

We suspected that the cause of jaw claudication was right atherothrombotic ECA occlusion and expected that collateral blood supply would ameliorate the jaw claudication over time. We did not choose interventional treatment since there were no physical findings of facial and lingual necrosis that required urgent revascularization and we expected improvement in the symptom over time, as the collateral circulation developed. Meanwhile, we initiated medication therapy, cilostazol, for antiplatelet action and peripheral vasodilatation.

**Figure 3:**
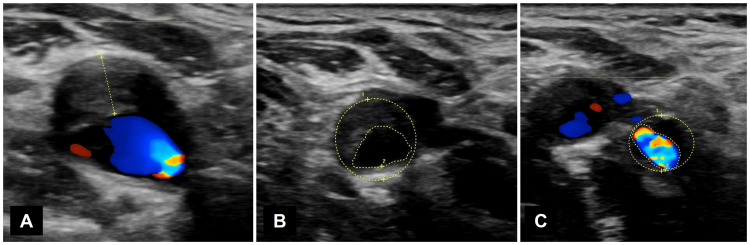
Carotid ultrasonography six months after the first visit showing atheromatous stenosis in the right internal carotid artery (A,B) and external carotid artery (C)

The jaw claudication gradually improved over time. He was able to consume small amounts of solid food within one month by slowly chewing it. In two months, the pain had substantially reduced, and the right superficial temporal artery (STA) in which the pulse was initially absent became weakly palpable. The pulsations of both STA were almost equal in three months. In four months, MR angiography showed recanalization of the stenosed right ECA near the bifurcation (Figure 2). The jaw claudication completely disappeared in six months. During this period, carotid ultrasonography showed plaques extending from the right common carotid artery to the ICA and ECA (Figure 3). Applying the European Carotid Surgery Trial method, 54% stenosis was detected in the right ICA. The peak systolic velocities of the right ECA and ICA were 2.98 m/s and 1.65 m/s, respectively. No abnormalities were observed in the left carotid artery. The patient remained asymptomatic until the last follow-up visit.

## Discussion

We encountered a patient with jaw claudication caused by ECA occlusion. Carotid ultrasonography and time-course changes in the MR images suggested ECA atherothrombosis, and no other embolic source was observed. The symptom improved not by the development of collateral supply, but by spontaneous ECA recanalization.

The most common cause of jaw claudication is GCA, whereas there are very few case reports of jaw claudication caused by atherothrombotic ECA occlusion (Table 1) [1,6-11]. Elevated levels of C-reactive protein and ESR are clinical features of GCA with high frequency [12]. Since visual disturbances, absent or diminished temporal artery pulse, and transient ischemic attack can occur in both atherothrombotic ECA occlusion and GCA [12], they are not specific to atherothrombotic ECA occlusion. In our experience, a combination of laboratory and multimodality examinations, such as ultrasonography, contrast-enhanced CT, and MR angiography, was helpful for the diagnosis. In addition to ECA stenosis, MR angiography and ultrasonography showed ICA stenosis. The manifestations of ECA ischemia, such as facial pain, can be a predictor of ICA occlusion [13]. Therefore, we must be careful not to overlook or underestimate facial and jaw pain, which can be a warning sign of a future stroke.

**Table 1:** Summary of previous reports on jaw claudication caused by atherosclerotic external carotid artery lesion ECA: external carotid artery, ICA: internal carotid artery, CEA: carotid endarterectomy, PTA: percutaneous transluminal angioplasty

Study	Lesion location	Imaging findings	Treatment
Lewis et al. (1978) [6]	ECA	Occlusion	Conservative treatment (aspirin, dipyridamole)
Argentino et al. (1980) [7]	ECA	Occlusion	CEA
Venna et al. (1986) [8]	ECA	Occlusion	CEA
Webster et al. (1994) [9]	ECA, ICA	>90% stenosis	CEA
Schiller et al. (2007) [11]	ECA	Severe stenosis	PTA
Janssens et al. (2008) [10]	ECA	Severe stenosis	CEA
Chen et al. (2011) [1]	ECA	Near occlusion	CEA

Jaw claudication caused by ECA occlusion can persist from 10 days to nine months with conservative management, and it improves spontaneously [1,3,6]. In conservative treatment, the development of collateral circulation is the predicted mechanism for symptomatic relief [6], and there is a previous report on jaw claudication that improved despite failure to recanalize the ECA [1]. In the present case, jaw claudication was gradually relieved and disappeared within six months through conservative treatment. The mechanism of symptomatic improvement was not due to the development of collateral circulation, but spontaneous ECA recanalization.

As described above, since jaw claudication caused by ECA occlusion often disappears over time, we believe that conservative treatment can be the first-line treatment. However, early interventional treatment is better in certain situations. Signs of facial or lingual necrosis require early interventional recanalization of the causative occlusion vessel [4,14]. Carotid endarterectomy (CEA) and percutaneous transluminal angioplasty (PTA) have been reported as effective treatments [1,4,7,11,14]. CEA was effective in ECA stenosis combined with symptomatic ICA stenosis, causing amaurosis fugax and transient ischemic attack [1]. Schiller et al. [11] reported that staged bilateral PTA was effective in ECA-confined bilateral stenosis with jaw claudication. In contrast, Janssens et al. [10] reported that the unilateral CEA ameliorated bilateral jaw claudication with bilateral ECA stenosis and suggested that even unilateral intervention could relieve bilateral ischemia. Thus, some cases require interventional treatment, but the algorithm of surgical management is still debatable.

## Conclusions

Although rare, we should consider not only GCA but also atherothrombotic ECA occlusion as a cause of jaw claudication. In this case, the symptom gradually disappeared in six months with spontaneous ECA recanalization by conservative therapy. Further investigation on a large number of cases is required to establish evidence-based treatment strategies.

## References

[1] Chen H, Kougias P, Lin PH, Bechara CF (2011). Jaw claudication in the era of carotid stenting. J Vasc Surg.

[2] de Norman JE (1970). Facial pain and vascular disease. Some clinical observations. Br J Oral Surg.

[3] Willfort-Ehringer A, Ahmadi R, Gruber D (2003). Effect of carotid artery stenting on the external carotid artery. J Vasc Surg.

[4] Domanin M, Isalberti M, Romagnoli S, Rolli A, Sommaruga S (2017). Acute hemifacial ischemia as a late complication of carotid stenting. J Vasc Surg Cases Innov Tech.

[5] Goodman BW Jr, Shepard FA (1983). Jaw claudication. Its value as a diagnostic clue. Postgrad Med.

[6] Lewis RR, Beasley MG, MacLean KS (1978). Occlusion of external carotid artery causing intermittent claudication of the masseter. Br Med J.

[7] Argentino C, Iadecola C, Pistolese GR, Faraglia V (1980). Ischaemic intermittent claudication of the masticatory muscles: two case reports. Ital J Neurol Sci.

[8] Venna N, Goldman R, Tilak S, Sabin TD (1986). Temporal arteritis-like presentation of carotid atherosclerosis. Stroke.

[9] Webster G, Beynon HL, Walport MJ (1994). Jaw claudication and amaurosis fugax secondary to atheromatous disease of the carotid arteries. Br J Rheumatol.

[10] Janssens MA, Van Thielen TH, Van Veer HG (2008). Jaw claudication as a result of carotid artery disease. Acta Chir Belg.

[11] Schiller A, Schwarz U, Schuknecht B, Mayer D, Hess K, Baumgartner RW (2007). Successful treatment of cold-induced neck pain and jaw claudication with revascularization of severe atherosclerotic external carotid artery stenoses. J Endovasc Ther.

[12] Ponte C, Martins-Martinho J, Luqmani RA (2020). Diagnosis of giant cell arteritis. Rheumatology (Oxford).

[13] Herishanu Y, Bendheim P, Dolberg M (1974). External carotid occlusive disease as a cause of facial pain. J Neurol Neurosurg Psychiatry.

[14] Bjordahl PM, Ammar AD (2011). Tongue necrosis as an unusual presentation of carotid artery stenosis. J Vasc Surg.

